# Effect of Saponification Condition on the Morphology and Diameter of the Electrospun Poly(vinyl acetate) Nanofibers for the Fabrication of Poly(vinyl alcohol) Nanofiber Mats

**DOI:** 10.3390/polym8100376

**Published:** 2016-10-21

**Authors:** Seong Baek Yang, Jong Won Kim, Jeong Hyun Yeum

**Affiliations:** 1 Department of Bio-fibers and Materials Science, Kyungpook National University, Daegu 41566, Korea; ysb@knu.ac.kr; 2 Department of Textile Engineering & Technology, Yeungnam University, Gyeongsan 38541, Korea

**Keywords:** electrospinning, heterogeneous saponification, poly(vinyl alcohol), poly(vinyl acetate), nanofiber mats

## Abstract

Novel poly(vinyl alcohol) (PVA) nanofiber mats were prepared for the first time through heterogeneous saponification of electrospun poly(vinyl acetate) (PVAc) nanofibers. The effect of varying the saponification conditions, including temperature, time, and concentration of the alkaline solution, on the morphology of the saponified PVA fibers were evaluated by field-emission scanning electron microscopy. At 25 °C, the saponified PVA fibers exhibited a broad diameter distribution. The average fiber diameter, however, was found to decrease with increasing saponification temperature. When the saponification time was increased from 6 to 30 h, the average fiber diameter decreased gradually from 1540 to 1060 nm. In addition, the fiber diameter and morphology were also affected by the concentration of the alkaline saponification solution. The most optimal conditions for fabrication of thin, uniform, and smooth PVA nanofibers corresponded to an alkaline solution containing 10 g each of NaOH, Na_2_SO_4_, and methanol per 100 g of water, a temperature of 25 °C, and a saponification time of 24 h.

## 1. Introduction

Unlike most vinyl polymers, poly(vinyl alcohol) (PVA) cannot be prepared by direct polymerization of the corresponding monomer as a result of its keto-enol tautomerism [1,2]. Instead, PVA is synthesized by polymerizing vinyl acetate, followed by conversion of the resulting poly(vinyl acetate) (PVAc) into PVA through saponification [3,4,5]. PVA nanofibers are currently used as drug [6] and protein carriers [7] as well as nanocomposite electrospun fiber matrices for antibacterial treatments [8], tissue engineering [9], and other biomedical applications [10]. Several research groups have used heterogeneous saponification to manufacture PVA from PVAc. For example, Lee et al. prepared PVA/PVAc microspheres through heterogeneous surface saponification of PVAc microspheres and investigated the rate of saponification in the presence of various ions [11]. In another study, Lee et al. developed a novel method of manufacturing syndiotactic PVA/poly(vinyl pivalate/vinyl acetate) microspheres through heterogeneous saponification and also investigated the effect of their initial diameter on the degree of saponification (DS) of PVA [12]. A simple, continuous heterogeneous saponification method was applied by Lyoo et al. to obtain high-molecular-weight PVA from suspension-polymerized PVAc, and the effect of saponification time and temperature on their DS was investigated [13]. In addition, the same group also prepared PVA/PVAc shell/core microspheres of different sizes by surface saponification of emulsion-polymerized PVAc [14].

In this study, heterogeneous saponification is used to convert PVAc into PVA. Previously, we reported the synthesis of PVAc/PVA/montmorillonite (MMT) nanocomposite microspheres by suspension polymerization and heterogeneous saponification and described the effect of the MMT presence on the PVAc polymerization and saponification rates [15]. In another report, we examined a novel method of manufacturing PVA/clay microspheres through suspension polymerization and heterogeneous saponification, where the formation of fully saponified nanocomposite microspheres was confirmed by proton nuclear magnetic resonance (^1^H-NMR) spectroscopy [16]. Many researchers have reported PVA nanofibers prepared by electrospinning of a PVA solution. Bai et al. demonstrated an easy and effective electrospinning technique for fabricating PVA/Au composite nanofibers [17]. In another study, the preparation of PVA/multi-walled carbon nanotubes was explored [18]. The effect of molecular weight on the fiber structure of electrospun PVA has been investigated by Koski et al. [19]. The preparation of electrospun PVA mats and their morphologies were studied by Zhang et al. [20], while the effect of pH on the morphology and the average diameter of electrospun PVA fibers was discussed by Son et al. [21]. Many researchers have also described cell propagation in modified nanofibers. Zhang et al. [22] examined nanofiber-modified surface directed cell movement and arrangement in a microsystem. The effect of the nanofiber arrangement in electrospun nanofibrous scaffolds on cell growth and elastin appearance of muscle cells was reported by Zhong et al. [23]. Surface-modified nanofibers have some benefits as a result of the larger specific surface area compared to smooth surface nanofibers. Recently, our group succeeded in converting PVAc nanofibers into PVA nanofibers using heterogeneous saponification technique. However, this work reported only the preparation of PVA nanofibers, without examining the effect and the most optimal nature of saponification conditions [24]. The present study aims to fabricate PVA nanofiber mats with a potential application in bio-materials, for example in animal testing, cell proliferation, and cell culture, and to investigate further the nanofiber formation.

In this work, we demonstrate an inexpensive technique for preparing PVA nanofiber mats through heterogeneous saponification of electrospun PVAc nanofibers, without the need for a PVA solution. In addition, the effect of saponification conditions on the morphology and diameter of the electrospun saponified PVAc nanofibers is investigated. The results showed that the optimal conditions for fabricating thin, uniform, and smooth saponified PVA nanofibers corresponded to an alkaline solution containing NaOH (10 g), Na_2_SO_4_ (10 g), and methanol (MeOH, 10 g) per 100 g of water, at a temperature of 25 °C, and a saponification time of 24 h.

## 2. Materials and Methods

### 2.1. Materials

Vinyl acetate (VAc) (Sigma-Aldrich, St. Louis, MO, USA) was purified by treatment with an aqueous solution of NaHSO_4_, followed by drying over anhydrous CaCl_2_ and distillation under N_2_ atmosphere. The PVA suspending agent with an average molecular weight of 127,000 and DS of 88% (Sigma-Aldrich) was recrystallized twice from MeOH before use. An aqueous alkaline solution containing NaOH (Duksan, Ansan, Korea), Na_2_SO_4_ (Duksan), and MeOH (Duksan) was prepared for heterogeneous saponification. Deionized water was utilized in all experiments.

### 2.2. Preparation of Poly(vinyl acetate) (PVAc) by Suspension Polymerization of vinyl acetate (VAc)

To prepare PVAc, the PVA suspending agent was dissolved in continuously stirred distilled water (120 mL) in a 250 mL reactor equipped with a condenser. The VAc monomer and 2,2′-azobis(2,4-dimethylvaleronitrile) (ADMVN) initiator were degassed and added to this mixture at a polymerization temperature of 15 °C, and the reaction temperature was increased to 60 °C. After a specified time period elapsed, the reaction was stopped and the resulting mixture was left for 1 h to isolate the obtained spherical PVAc particles. After a prearranged time has elapsed, the reaction mixture was cooled and set aside for 1 day to allow the precipitation and partition of the PVAc particles. After this time, the accumulated PVAc particles were washed with warm water. Gravimetric analysis confirmed the successful conversion of the VAc monomer [16]. An overview of the polymerization conditions is provided in Table 1.

### 2.3. Electrospinning of PVAc Nanofibers

In order to prepare an electrospinning solution, the manufactured PVAc was dissolved over 2 h in MeOH at room temperature, with continuous stirring. All experiments in this work employed PVAc at 15 wt % (a concentration based on the weight of the solution). During electrospinning, a high voltage of 15 kV (CPS-60K02, VIT, Chungpa EMT Co., Ltd., Seoul, Korea) was applied to an alligator clip attached to a syringe needle containing 30 mL of the PVAc solution. The solution was transported to the blunt needle using a syringe pump to control the flow rate. An electrically grounded piece of Al foil, placed at a vertical distance of 15 cm from the needle tip, was used to collect the obtained fibers, which were subsequently air-dried and removed from the Al foil before saponification. These spinning conditions were found to be the most optimal for manufacturing of PVAc mats.

### 2.4. Heterogeneous Saponification of PVAc Nanofibers

To convert the PVAc nanofibers into PVA nanofibers, heterogeneous saponification was performed in a flask equipped simultaneously with a thermocouple, a reflux condenser, a dropping funnel, and a stirring device. An alkaline solution for the saponification process was prepared by adding NaOH (5–10 g), Na_2_SO_4_ (5–10 g), and MeOH (5–10 g) to deionized water (100 g). In our previous work, we found that the optimum composition of the saponification solution consists of NaOH (10 g), Na_2_SO_4_ (10 g), and MeOH (10 g) added to deionized water (100 g) [24]. If more than 10 g of NaOH were used, the saponified PVA nanofibers exhibited a yellow color. Therefore, we excluded such harsh conditions from this study (i.e., above 10 g of NaOH). To investigate the effect of changing the saponification solution, we specifically tested a solution prepared using NaOH (5 g), Na_2_SO_4_ (5 g), and MeOH (5 g) in deionized water (100 g). Subsequently, the electrospun PVAc fibers were slowly added to the prepared alkaline solution at various temperatures (25, 35, and 45 °C), employing moderate stirring. The PVA nanofibers were formed from the PVAc fibers after different saponification times (6, 12, 18, 24, and 30 h). The obtained alkaline solution mixture was poured into cold water for 1 min in order to precipitate the resulting PVA nanofiber mats, which were subsequently washed several times with deionized water and dried under vacuum for 24 h. The complete saponification conditions are recorded in Table 2.

### 2.5. Characterization

The molecular weights of the electrospun PVA nanofibers and the fully saponified PVA nanofibers were determined using gel permeation chromatography (GPC, Waters, Milford, MA, USA) [25]. The surface morphology of the saponified PVA nanofiber mats was studied using field-emission scanning electron microscopy (FE-SEM, SU8220, Hitachi, Tokyo, Japan), and their DS values were determined using a ^1^H-NMR spectrometer (AVANCE III 500, Bruker, Karlsruhe, Germany). A Fourier transform infrared (FT-IR) spectrometer (Frontier, Perkin Elmer, Waltham, MA, USA) was utilized to measure the conversion of PVAc nanofibers to PVA nanofibers. Fiber diameters were evaluated from the obtained FE-SEM images using the Photoshop CS 7 software. The analysis examined at least 20 different fibers and 100 different randomly selected segments from each image. The tensile strength was measured using the ZWICK Z005 testing machine (Zwick GmbH, Ulm, Germany).

## 3. Results and Discussion

**Figure 1:**
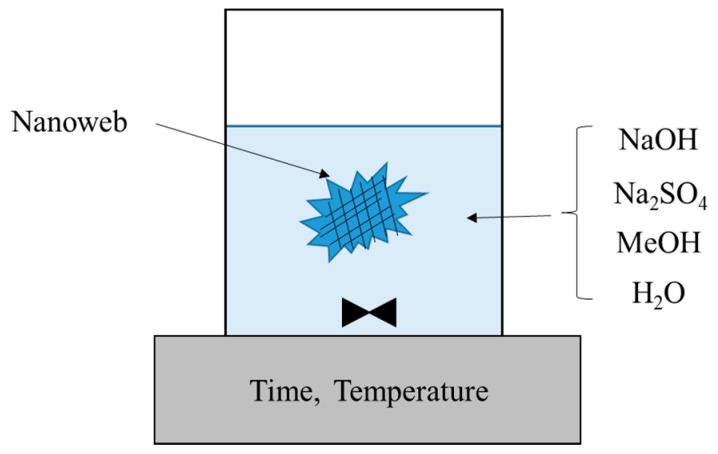
Schematic illustration of the heterogeneous saponification process employed for manufacturing of PVA nanofiber mats.

Owing to the various advantages of PVA polymers, many research groups have attempted to fabricate electrospun PVA bulk nanofiber mats. In this work, we have prepared for the first time saponified PVA nanofiber mats from PVAc nanofibers through heterogeneous saponification. Figure 1 shows a schematic illustration of the heterogeneous saponification process employed in the synthesis of PVA nanofiber mats from PVAc nanofibers. The morphology of the saponified PVA nanofiber mats was found to vary depending on the different saponification conditions, such as the temperature, time, and the concentration of the saponification solution (i.e., the alkaline solution).

**Figure 2:**
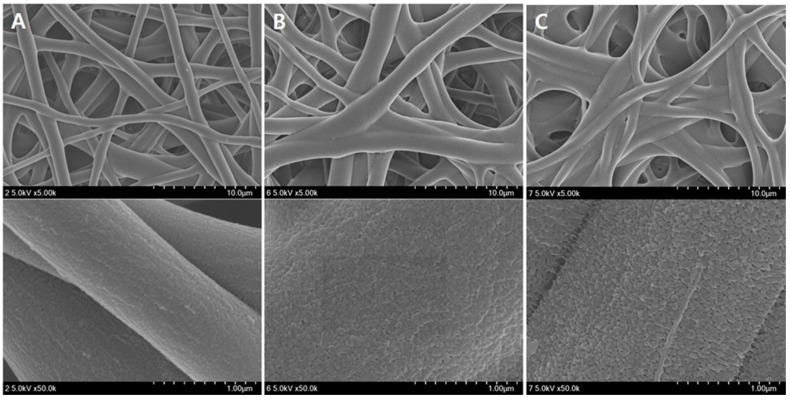
Field emission scanning electron microscope (FE-SEM) images of saponified PVA nanofiber mats obtained at different temperatures: (**A**) 25 °C; (**B**) 35 °C; and (**C**) 45 °C. The quantities of the reagents in the saponification solution (NaOH = 10 g, Na_2_SO_4_ = 10 g, MeOH = 10 g, and H_2_O = 100 g) and the reaction time (12 h) were kept constant.

**Figure 3:**
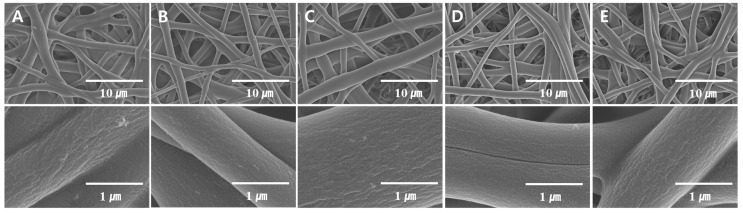
FE-SEM images of saponified PVA nanofiber mats obtained at different saponification times: (**A**) 6 h; (**B**) 12 h; (**C**) 18 h; (**D**) 24 h; and (**E**) 30 h. The quantities of the reagents in the saponification solution (NaOH = 10 g, Na_2_SO_4_ = 10 g, MeOH = 10 g, and H_2_O = 10 g) and the reaction temperature were kept constant (25 °C).

Figure 2 shows the FE-SEM images of the PVA nanofiber mats produced by heterogeneous saponification of PVAc nanofibers for 12 h at different temperatures (25, 35, and 45 °C), employing an alkaline solution containing 10 g each of NaOH, Na_2_SO_4_, and MeOH added to 100 g of H_2_O. We found that 25 °C was the optimal temperature for obtaining thin and uniform saponified PVA nanofibers with a smooth surface. Figure 3 presents the FE-SEM images of PVA nanofibers produced by heterogeneous saponification of PVAc nanofibers at various times (6, 12, 18, 24, and 30 h), at a temperature of 25 °C, and a constant alkaline solution concentration corresponding to 10 g of NaOH, 10 g of Na_2_SO_4_, and 10 g of MeOH per 100 g of H_2_O. The obtained results indicate that the saponification time affects the fiber morphology significantly. Specifically, comparatively smooth, thin, and uniform saponified PVA fibers with small diameter were easily obtained after 24 h saponification time (Figure 3D). In contrast, such fibers were not observed at shorter saponification times of 6 or 18 h (see Figure 3A,C, respectively). Furthermore, rough surface fibers with a small diameter were produced when the saponification time was increased to 30 h (Figure 3E).

**Figure 4:**
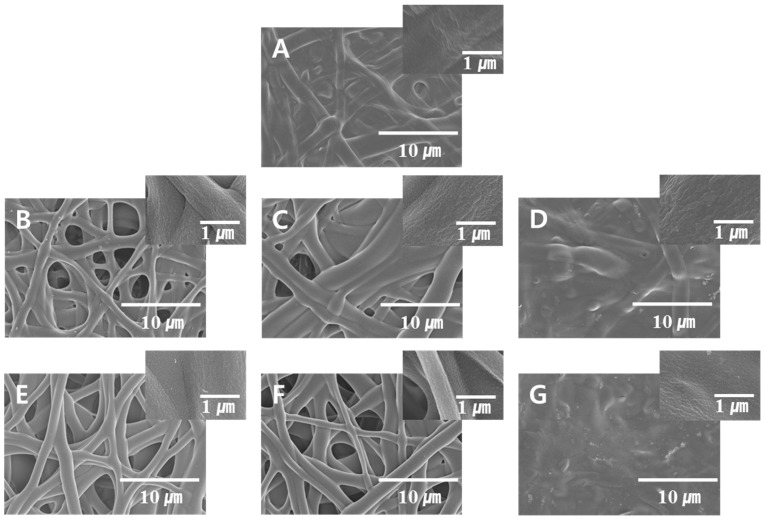
FE-SEM images of saponified PVA nanofiber mats obtained using saponification solution prepared using different respective quantities of NaOH, Na_2_SO_4_, and MeOH per 100 g of H_2_O: (**A**) 5, 5, and 5 g; (**B**) 7.5, 5, and 5 g; (**C**) 5, 7.5, and 5 g; (**D**) 5, 5, and 7.5 g; (**E**) 10, 5, and 5 g; (**F**) 5, 10, and 5 g; and (**G**) 5, 5, and 10 g. The saponification temperature and time were kept constant at 25 °C and 24 h, respectively.

Next, a series of experiments were conducted using different concentrations of saponification solution reagents and a constant temperature and time of 25 °C and 24 h, respectively. The results presented in Figure 4 indicate that the fiber morphology depends strongly on the ratio of the saponification solutions constituents (NaOH, Na_2_SO_4_, and MeOH). After a careful comparison of the FE-SEM images displayed in Figure 4, we were able to conclude that preparation of saponified PVA nanofibers at low concentrations of NaOH and Na_2_SO_4_ is not possible (see Figure 4A,C,D,G). On the other hand, the saponified fibers could be easily obtained at high concentrations of NaOH and Na_2_SO_4_ (Figure 4E,F). The presence of MeOH also affected the morphology of the saponified PVA nanofibers significantly (Figure 4A,D,G). While saponified PVA was unable to retain its fibrous morphology at high MeOH concentrations (Figure 4D,G), this effect of MeOH addition on the fiber morphology became insignificant at higher concentrations of NaOH and Na_2_SO_4_ (Figure 3D).

**Figure 5:**
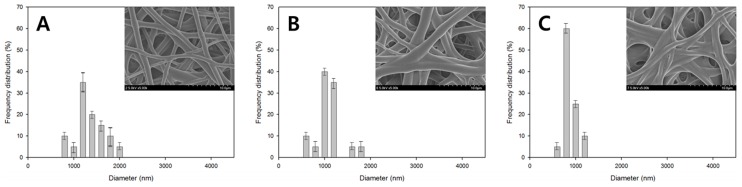
Fiber diameter histogram plots and FE-SEM images for saponified PVA nanofiber mats obtained at different saponification temperatures: (**A**) 25 °C; (**B**) 35 °C; and (**C**) 45 °C. The saponification solution contained NaOH (10 g), Na_2_SO_4_ (10 g), and MeOH (10 g) in H_2_O (100 g) in each case. The time was kept constant at 12 h.

**Figure 6:**
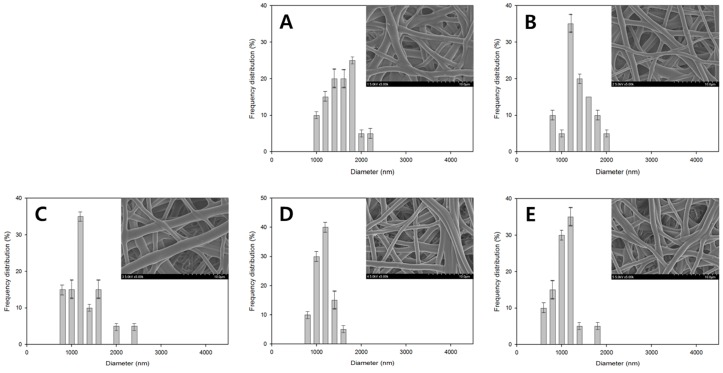
Fiber diameter histogram plots and FE-SEM images for saponified PVA nanofiber mats obtained at different saponification times: (**A**) 6 h; (**B**) 12 h; (**C**) 18 h; (**D**) 24 h; and (**E**) 30 h. The saponification solution was prepared using the following reagents: NaOH (10 g), Na_2_SO_4_ (10 g), and MeOH (10 g) per 100 g of H_2_O. The temperature was kept constant at 25 °C.

**Figure 7:**
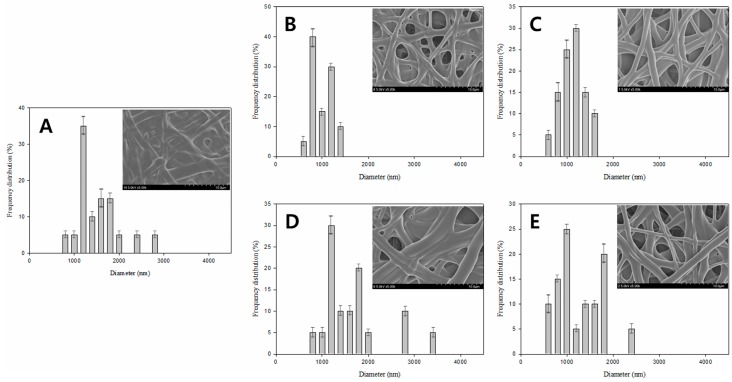
Fiber diameter histogram plots and FE-SEM images for saponified PVA nanofiber mats obtained using a saponification solution prepared with different respective quantities of NaOH, Na_2_SO_4_, and MeOH per 100 g of H_2_O: (**A**) 5, 5, and 5 g; (**B**) 7.5, 5, and 5 g; (**C**) 10, 5, and 5 g; (**D**) 5, 7.5, and 5 g; and (**E**) 5, 10, and 5 g. The saponification time and temperature were kept constant at 24 h and 25 °C, respectively.

The fiber diameter distributions for the saponified PVA nanofibers produced at different saponification temperatures are shown in Figure 5. The obtained data revealed a decrease in the average fiber diameter with increasing saponification temperature. Large amounts of thin fibers with diameters below 1000 nm were observed at a saponification temperature of 45 °C. The fiber arrangement in samples prepared at this temperature, however, was not regular (Figure 5C). In contrast, regularly-arranged fibers with an average diameter of 1350 nm were obtained at 25 °C (Figure 5A). A narrow fiber diameter distribution was observed at the higher temperature of 45 °C, while at the lower temperature of 25 °C, the fiber diameter exhibited a broad distribution. Increasing the temperature (and thus the saponification rate) promotes the conversion of PVAc into PVA, which in turn stimulates the formation of thinner fibers since the PVA species formed during saponification can be better dissolved in the aqueous solution. The effect of saponification time on the diameter distribution of the saponified PVA nanofibers is shown in Figure 6. The diameter of the saponified PVA nanofibers was found to gradually decrease from 1540 to 1060 nm with increasing saponification time from 6 to 30 h. The longer saponification times increase the dissolution rate for the PVA surface layers in aqueous solutions, thereby decreasing the resulting fiber diameter (Figure 6E). These smaller diameter fibers are, however, characterized by a rough surface (Figure 3E). Therefore, the most optimal saponification time for the formation of PVAc nanofibers was found to be 24 h, which produces an average fiber diameter of 1150 nm (Figure 6D). Varying the concentrations of reagents in the saponification solution was also found to change the fiber diameter, as shown in Figure 7. With a saponification solution employing concentrations that correspond to 5 g of NaOH, 5 g of Na_2_SO_4_, and 5 g of MeOH per 100 g of H_2_O, the obtained fibers were essentially affixed to each other, and their average diameter was equal to 1520 nm (Figure 7A). However, the fiber morphology changed when the concentration of NaOH was increased to 10 g per 100 g of H_2_O. Specifically, the fiber structure changed from the united morphology to a segregated one, thus decreasing the corresponding fiber diameter (Figure 7C). On the other hand, the average fiber diameter obtained using 7.5 g of Na_2_SO_4_ (Figure 7D) was greater than that produced at 10 g of Na_2_SO_4_ (Figure 7E).

**Figure 8:**
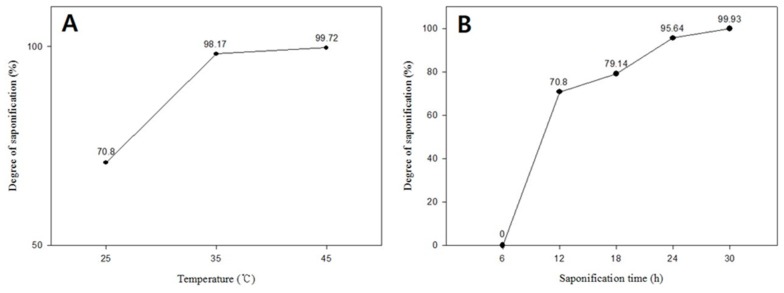
(**A**) The effect of saponification temperature on the degree of saponification (DS) (%) of PVA saponified for 12 h; (**B**) The effect of saponification time on the DS of PVA saponified at 25 °C. The concentration of the saponification solution (NaOH, Na_2_SO_4_, and MeOH at 10 g each per 100 g of H_2_O) was kept constant.

**Figure 9:**
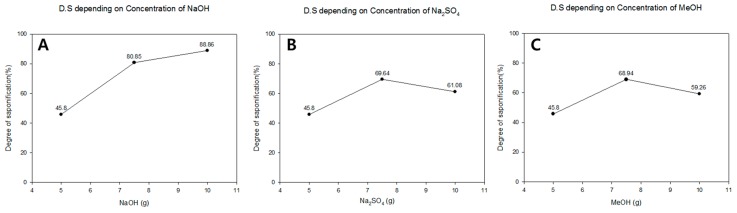
The effect of: (**A**) NaOH; (**B**) Na_2_SO_4_; and (**C**) MeOH concentration on the DS (%) of saponified PVA nanofibers. The saponification time and temperature were kept constant at 24 h and 25 °C, respectively.

The dependence of DS on the saponification temperature is shown in Figure 8A. The obtained DS value was much higher at 45 °C than those observed at 25 and 35 °C, most likely as a result of the accelerated rate of saponification at higher temperatures [13]. The DS of PVA prepared by heterogeneous saponification exceeded 99% at a saponification temperature of 45 °C. The corresponding average fiber diameter, however, was also larger at this temperature compared to that obtained at 25 and 35 °C (Figure 2). The dependence of DS on the saponification time at 25 °C is plotted in Figure 8B, showing that the DS increases with increasing length of saponification time. The maximum DS for the PVA nanofibers saponified for 30 h exceeded 99%. The effect of varying the quantity of reagents in the saponification solution (i.e., NaOH, Na_2_SO_4_, and MeOH per 100 g of H_2_O) on the DS of the saponified PVA nanofiber mats was also investigated in this work. The DS values for the saponified PVA at three different concentrations of NaOH are presented in Figure 9A, which shows an increase in the DS with increasing concentration of NaOH, reaching the highest DS value at a NaOH content of 10 g per 100 g of water. The DS dependence on the Na_2_SO_4_ concentration is shown in Figure 9B. The DS value was found to increase with increasing concentration of Na_2_SO_4_ up to 7.5 g per 100 g of water. Further increase in concentration resulted in a decrease in the value of DS. The maximum DS value was obtained at 7.5 g of Na_2_SO_4_, however, the formation of thin saponified nanofibers was no longer possible at this particular concentration of Na_2_SO_4_ (Figure 4C). Figure 9C shows the effect of changing the MeOH concentration on the DS of the saponified PVA nanofibers. The obtained dependence exhibits a trend similar to that observed for Na_2_SO_4_ (Figure 9B). The maximum DS value of over 68% is reached at a MeOH concentration of 7.5 g per 100 g of water (Figure 9C). The obtained fiber quality at this MeOH concentration, however, was not very good (Figure 4D).

**Figure 10:**
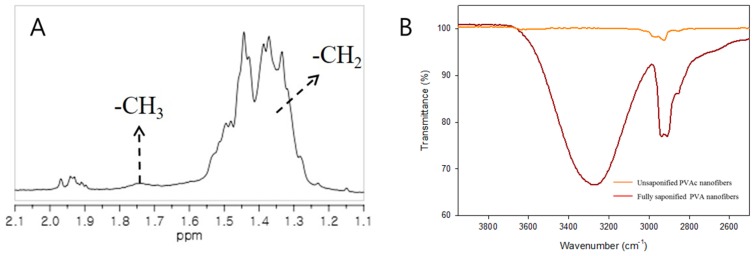
(**A**) A partial proton nuclear magnetic resonance spectrum of fully saponified PVA nanofibers; (**B**) Partial Fourier transform infrared spectroscopy spectra for un-saponified PVAc nanofibers (orange) and fully saponified PVA nanofibers (red). The polymer concentration was kept constant at 15 wt % and the saponification solution was comprised of NaOH (10 g), Na_2_SO_4_ (10 g), MeOH (10 g), and H_2_O (100 g). The saponification temperature was 25 °C, the saponification time was 30 h, and DS was 99.93%.

**Figure 11:**
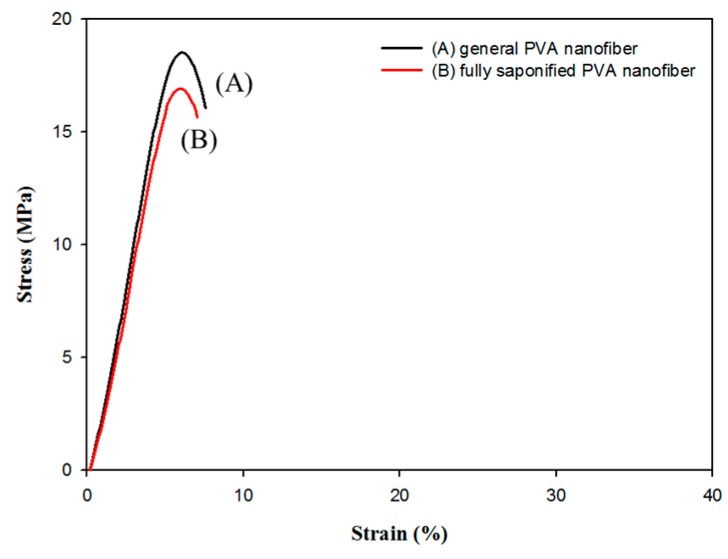
Stress-strain curve of: (**A**) general electrospun PVA nanofibers; and (**B**) fully saponified PVA nanofibers formed by heterogeneous saponification of electrospun PVAc nanofibers. The concentration of these nanofibers was at 15 wt % and the saponification solution was prepared using NaOH (10 g), Na_2_SO_4_ (10 g), MeOH (10 g) and H_2_O (100 g). The saponification temperature and time were 25 °C and 30 h, respectively. The DS value of the fully saponified nanofibers was 99.93%.

In this work, all saponified samples were characterized by ^1^H-NMR and Figure 10A shows the corresponding ^1^H-NMR data for the PVA nanofibers saponified for 30 h at 25 °C. The DS of the saponified PVA nanofibers can usually be calculated from the ratio of the area of the methyl group and the area of the methylene groups present in the ^1^H-NMR spectrum (appearing at 1.74 ppm and 1.4 ppm, respectively). However, no methyl peaks were observed in the spectra shown in Figure 10 indicating that all of the PVAc methyl groups have been converted into hydroxyl groups during saponification. Therefore, the NMR analysis confirmed that fully saponified PVA nanofibers can be obtained at saponification conditions corresponding to an alkaline solution containing 10 g each of NaOH, Na_2_SO_4_, and MeOH per 100 g of H_2_O, a temperature of 25 °C, and a saponification time of 30 h. The FT-IR spectra of both the un-saponified PVAc and saponified PVA nanofibers were measured as shown in Figure 10B. The vibrational bands at 2923 and 2865 cm^−1^ correspond to CH_3_ asymmetric stretch and PVAc symmetric stretch, respectively [26]. The broad band viewed in the 3600–3200 cm^−1^ region after the saponification process can be assigned to the O–H stretch, arising from both intermolecular and intramolecular hydrogen bonds. The vibration band in the 3000–2800 cm^−1^ region corresponds to the C–H alkyl group stretch [27], further demonstrating the successful formation of PVA nanofibers from PVAc nanofibers. The molecular weights of the fully saponified PVA nanofibers and general electrospun PVA nanofibers were obtained by GPC and are listed in Table 3. Examination of the tensile strength revealed that the tensile strength (Figure 11) of the electrospun PVA nanofibers is similar to that of the fully saponified PVA nanofibers. The results of the molecular weight determination and the tensile strength analysis indicated that the mechanical properties of fully saponified PVAc nanofibers do not deteriorate as a result of the heterogeneous saponification process.

## 4. Conclusions

In this study, completely saponified PVA nanofiber mats were obtained successfully through heterogeneous saponification of PVAc nanofibers, and the effect of saponification conditions, including temperature, saponification time, and the alkaline solution concentration on the morphology of the fabricated PVA nanofibers was evaluated. The obtained FESEM images demonstrated that the resulting fiber diameter decreases with increasing saponification time and temperature, while varying the concentration of the alkaline solution affected both the fiber diameter and morphology. In addition, the DS of PVA nanofibers was also found to depend on the saponification time, temperature, and alkaline solution concentration. The most optimal saponification conditions for the fabrication of thin, uniform, and smooth PVA nanofibers were found to correspond to an alkaline solution containing 10 g each of NaOH, Na_2_SO_4_, and MeOH per 100 g of H_2_O, a temperature of 25 °C, and a saponification time of 24 h.

## Figures and Tables

**Table 1:** Reaction conditions for the suspension polymerization of VAc.

Condition	Value
Type of initiator	ADMVN
Type of suspending agent	PVA
Initiator concentration	0.0001 mol/mol of VAc
Suspending agent concentration	1.5 g/dL of water
VAc/water	0.5 L/L
rpm	400
Temperature	60 °C

ADMVN: 2,2′-azobis(2,4-dimethylvaleronitrile); PVA: poly(vinyl alcohol); VAc: Vinyl acetate.

**Table 2:** The reagents and conditions employed in the heterogeneous saponification of PVAc nanofibers and the resulting degree of saponification. (DS: Degree of saponification).

Figure	NaOH (g)	Na_2_SO_4_ (g)	MeOH (g)	H_2_O (g)	Temparature (°C)	Time (h)	DS (%)
Figure 2A	10	10	10	100	25	12	70.8
Figure 2B	10	10	10	100	35	12	98.17
Figure 2C	10	10	10	100	45	12	99.72
Figure 3A	10	10	10	100	25	6	0
Figure 3B	10	10	10	100	25	12	70.8
Figure 3C	10	10	10	100	25	18	79.14
Figure 3D	10	10	10	100	25	24	95.64
Figure 3E	10	10	10	100	25	30	99.93
Figure 4A	5	5	5	100	25	24	45.8
Figure 4B	7.5	5	5	100	25	24	80.85
Figure 4C	5	7.5	5	100	25	24	88.86
Figure 4D	5	5	7.5	100	25	24	69.64
Figure 4E	10	5	5	100	25	24	61.08
Figure 4F	5	10	5	100	25	24	68.94
Figure 4G	5	5	10	100	25	24	59.26

**Table 3:** Molecular weights of generally electrospun PVA nanofibers and saponified PVA nanofibers with DS of 99.9% [24], as determined by gel permeation chromatography.

Sample	*M*_w_ (g/mol)	*M*_n_ (g/mol)	Polydispersity index (*M*_w_/*M*_n_)
General PVA nanofibers	82,125	10,841	7.57
Saponified PVA nanofibers	84,216	9,868	8.53
